# A206 SYNERGISTIC EFFECTS OF HISTAMINE AND PROTEASES IN VISCERAL HYPERSENSITIVITY

**DOI:** 10.1093/jcag/gwac036.206

**Published:** 2023-03-07

**Authors:** D A Gilmour, N Jimenez Vargas, A Omar, S Vanner, A Lomax, D Reed

## Abstract

**Background:**

Irritable bowel syndrome (IBS) is characterized by chronic abdominal pain. Previous work has shown IBS fecal supernatants (FS) increase the excitability of nociceptive neurons compared to healthy volunteer FS. Altered production of intestinal mediators, including elevated histamine content and proteolytic activity, have been implicated in these effects. We hypothesized that the effects of histamine and proteases on neuronal excitability may be synergistic.

**Purpose:**

1) Determine whether the excitatory effect of IBS patient FS can be abolished by histamine receptor antagonists and protease inhibitors. 2) Investigate whether the excitatory effects of histamine and proteases potentiate each other’s effects on visceral nociception

**Method:**

Extracellular recordings were performed, measuring action potentials from mechanosensitive extrinsic afferent nerves innervating the mouse colon during colonic distensions to 60 mmHg. Colons were perfused (20 minutes) with histamine receptor antagonists (1 µM pyrilamine, 10 µM ranitidine, 30 nM clobenpropit, 1 µM JNJ7777120) or protease inhibitors (10 µM EDTA, 0.2 µM bestatin, 0.03 µM Pepstatin A, 0.03 µM E-64) prior to treatment with the IBS patient FS. Perforated patch clamp experiments were used to measure neuronal excitability from mouse dorsal root ganglion neurons by recording rheobase (minimum current required to elicit an action potential fire). This technique was used to examine the effect of subthreshold concentrations of histamine (1µM) and trypsin (5nM), as well as their simultaneous administration on the neurons.

**Result(s):**

Luminal administration of histamine antagonists or protease inhibitors blocked the excitatory effect of the IBS patient FS (two-way ANOVA P=0.44 for protease antagonists, two-way ANOVA P=0.37 for histamine antagonists). Patch clamp experiments revealed that a subthreshold concentration of either histamine (1µM) or trypsin (5nM) had no effect on neuronal rheobase, whereas the combination significantly decreased neuronal rheobase (one-way ANOVA with Tukey’s test, P=0.006).

**Image:**

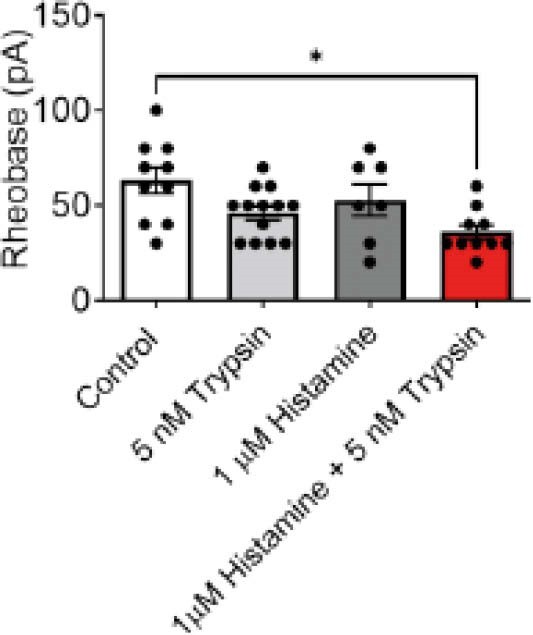

**Conclusion(s):**

These findings demonstrate that either histamine receptor antagonists or protease inhibitors inhibit the excitatory effect of IBS patient FS and suggest that a potentiating effect exists between the actions of histamine and proteases on nociceptive neurons.

**Disclosure of Interest:**

None Declared

